# Technical Note: Facilitating Laparoscopic Liver Biopsy by the Use of a Single-Handed Disposable Core Biopsy Needle

**DOI:** 10.1155/2013/462498

**Published:** 2013-04-16

**Authors:** M. I. Trochsler, Q. Ralph, F. Bridgewater, H. Kanhere, Guy J. Maddern

**Affiliations:** ^1^University of Adelaide Discipline of Surgery, The Queen Elizabeth Hospital, 28 Woodville Road, Woodville, SA 5011, Australia; ^2^Division of Surgery, The Queen Elizabeth Hospital, Woodville, SA 5011, Australia; ^3^Port Lincoln Hospital, Oxford Terrace, Port Lincoln, SA 5606, Australia

## Abstract

Despite the use of advanced radiological investigations, some liver lesions cannot be definitely diagnosed without a biopsy and histological examination. Laparoscopic Tru-Cut biopsy of the liver lesion is the preferred approach to achieve a good sample for histology. The mechanism of a Tru-Cut biopsy needle needs the use of both hands to load and fire the needle. This restricts the ability of the surgeon to direct the needle into the lesion utilising the laparoscopic ultrasound probe. We report a technique of laparoscopic liver biopsy using a disposable core biopsy instrument (BARD (R) disposable core biopsy needle) that can be used single-handedly. The needle can be positioned with laparoscopic graspers in order to reach posterior and superior lesions. This technique can easily be used in conjunction with laparoscopic ultrasound.

## 1. Introduction

Increased availability of ultrasound, computed tomography (CT), and magnetic resonance imaging (MRI) has resulted in incidental hepatic masses being reported more frequently. Indeterminate lesions especially in the cirrhotic liver often pose a diagnostic challenge. Specific radiological features such as a central scar in focal nodular hyperplasia or venous washout in hepatocellular carcinoma (HCC) are not always present. Colli et al. estimated 68% sensitivity and 93% specificity of spiral CT in diagnosing HCC compared with pathologic examination in their systematic review [1]. In a small number of cases—even with the use of 4-phase multidetector CT and contrast-enhanced MRI—a conclusive answer as to whether the lesion is benign or malignant or has a malignant potential might not be possible [2]. 

Laparoscopic Tru-Cut biopsy provides a definitive approach to determine the nature of a liver lesion. Percutaneous biopsy carries the risk of needle track or peritoneal seeding especially in the setting of a hepatocellular carcinoma [3, 4]. Laparoscopy and laparoscopic liver biopsy present an alternative and allows assessment of the peritoneal cavity to exclude advanced disease and gross liver cirrhosis in the same sitting. 

Lesions in the superior (2, 4a, 8) and posterior (6, 7) segments of the liver are technically challenging to biopsy during laparoscopy. A low lying and shallow rib cage combined with location of the lesions in the superior liver segments further increases the technical difficulty of laparoscopic liver biopsy. The biopsy needle may need to be introduced through the abdominal wall angled cephalad in order to reach the superior segments. 

A Tru-Cut biopsy needle often lacks the length to reach the lesion in this situation. Further, the Tru-Cut needle cannot be used with one hand. This restricts the surgeon's ability to operate another instrument (e.g., grasper, diathermy, or argon probe) at the same time and makes their use difficult in combination with laparoscopic ultrasound.

We report a technique of laparoscopic liver biopsy using a disposable core biopsy instrument (BARD (R) disposable core biopsy needle) that can be used single handedly. The needle may also be directed with a laparoscopic grasper in order to reach superior and posterior lesions. This technique can be easily used in conjunction with laparoscopic ultrasound.

## 2. Case Report

**Figure 1:**
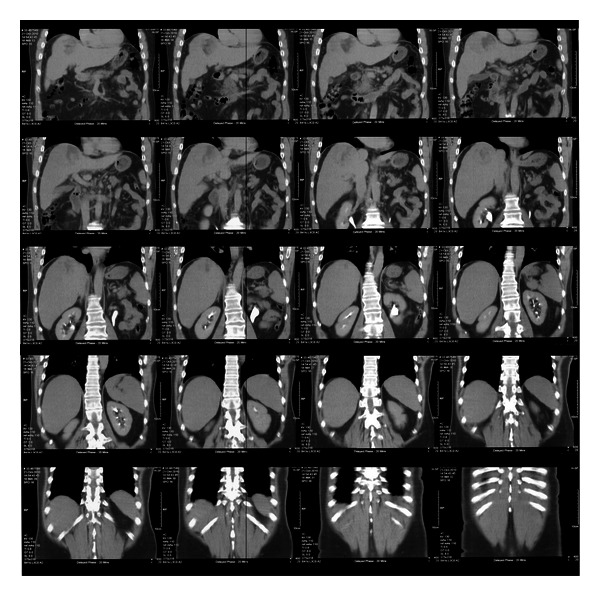
Abdominal CT scan with portovenous contrast showing a lesion in segment 7/8 of the liver.

A 61-year-old male patient was under surveillance for hepatitis B. He had a history of moderate daily alcohol intake. A routine ultrasound of his liver revealed a new lesion in segment 8 of the liver on the background of cirrhotic changes. Hepatic function was preserved (Child-Pugh class A). A subsequent abdominal CT scan confirmed a heterogeneous mass in segment 7/8 of the liver, with regions of central necrosis and extension to the capsular surface (Figure 1). Carcinoembryonic antigen and alpha fetoprotein serum levels were within normal limits. A surgical consult to determine a tissue diagnosis and resectability was sought. 

Multidisciplinary consensus concluded that the mass was resectable. On the background of hepatitis B, regular alcohol intake, and imaging findings consistent with possible cirrhotic changes throughout the liver, a decision to further assess the liver by laparoscopy and perform a laparoscopic biopsy of the mass in segment 8 as well as the “normal” liver tissue was made.

Intraoperatively the liver showed macro nodular changes over the entire surface. The liver was rigid and nonpliable. There was no evidence of disseminated disease within the abdominal cavity. Several tissue biopsies of the lesion in segment 8 were obtained utilizing the technique described below. The biopsy of “normal” liver tissue was obtained from segment 3 to establish the microscopic extent of cirrhosis. 

The histology revealed a moderately differentiated hepatocellular carcinoma in the right-sided liver lesion, as well as the serendipitous finding of another minute focus of a hepatocellular carcinoma in the biopsy from segment III. Further, severe focal fibrosis suggesting early development of cirrhosis with moderate activity was found. 

These findings precluded the patient from resectional surgery on the basis of bilateral hepatocellular carcinoma with extensive liver cirrhosis. The patient was referred for alternative treatment with an established tissue diagnosis of the liver lesion and proof of liver cirrhosis. 

## 3. Technique for Liver Biopsy

**Figure 2:**
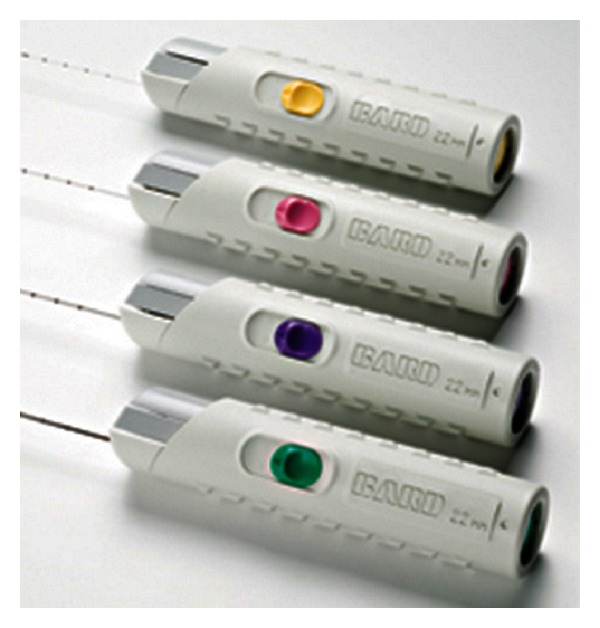
BARD disposable core biopsy needles.

BARD (R) disposable core biopsy needles (MC 1820, MC 1825), length 20/25 cm, diameter 18 G, were used (Figure 2). These biopsy needles are available in a range of lengths and diameters depending on the surgeon's preference and anatomical situation.

The patient is placed in a supine position under general anesthesia. The surgeon and the assistant stand on the patient's left side. A periumbilical open technique is used to gain access to the abdomen. A 10/12 mm laparoscopic port is inserted and a pneumoperitoneum is created. Two additional 5 mm ports are inserted; one in the epigastrium slightly to the left of the midline and another in the right upper quadrant. The position of these ports can be varied depending on the location of the lesion. If a laparoscopic ultrasound is required to locate parenchymal tumors, the right-sided 5 mm port is replaced with a 10/12 mm port. In selected cases, only one additional 5 mm port might be sufficient. 

**Figure 3:**
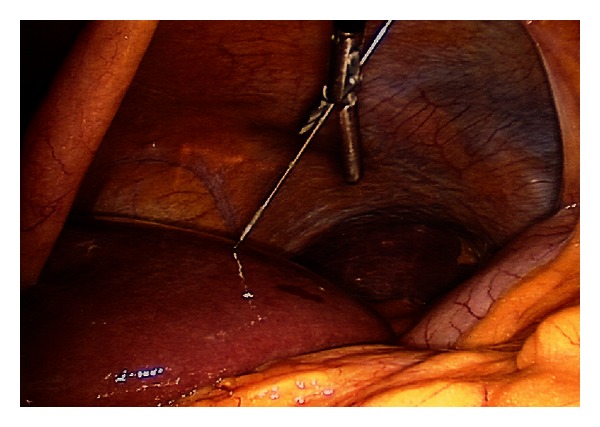
Guidance of the core biopsy needle using a laparoscopic grasper.

After inspection of the peritoneal cavity, the table is placed in a reverse Trendelenburg and rotated left side down. This facilitates the exposure of the right lateral and superior segments of the liver. The BARD (R) disposable core biopsy needle is introduced percutaneously or via an additional port into the abdomen from a point 1-2 cm inferior to the costal margin and in line with the liver lesion. The needle is directed towards the lesion. This can be facilitated by guidance of the needle with a blunt grasper in the surgeon's nondominant hand within the abdominal cavity (Figure 3). An argon probe is introduced into the abdomen by the assistant using the second 5 mm port. This is positioned in close proximity of the biopsy site to quickly and effectively control any bleeding from this area. 

The disposable core biopsy needle can now be inserted into the lesion. The instrument allows single-handed release of the biopsy mechanism without having to reposition the instruments. Once the needle is withdrawn the assistant instantly coagulates the biopsy site with the argon probe. This procedure can be repeated several times with the same core biopsy needle. We recommend at least two passes per lesion and if indicated a baseline biopsy of the “normal” liver tissue. 

Hemostasis is confirmed by evacuating the pneumoperitoneum under vision. The abdominal wall fascia is closed at the 10/12 mm port sites. 

## 4. Discussion

This technique allows safe and efficient laparoscopic biopsies of liver lesions for diagnostic purposes. There are benefits for both the surgeon and the patient. 

The BARD (R) disposable core biopsy needle is a reliable instrument which can be used single handedly. The needle is preloaded by retracting the spring loading mechanism with a retractable C-shaped button on the handle of the needle. The tip of the instrument is then simply introduced into the targeted lesion, and either one of the two buttons on the handle releases the biopsy mechanism. This “fires” the needle into the lesion in a manner similar to a Tru-Cut needle and reliably produces excellent cores for histopathology. The thin design of the needle allows the tip to be directed by applying lateral force along the shaft of the needle. There is a range of needle lengths and calibers available. In our experience, a biopsy needle 20 cm in length and 18 G caliber is sufficient to access most lesions.

The biggest advantage is the ability of the surgeon to utilize the nonoperating hand to use another instrument or laparoscopic ultrasound during the procedure.

It is possible for the surgeon to use a laparoscopic grasper to guide the needle into its target. Alternatively, the grasper might be used to manipulate the liver in the desired position or retract overlying omentum. A bowel grasper can be used to hold the liver edge in position for transverse liver biopsies. 

The procedure can be further simplified for superficial lesions in the anterior and inferior segments. In these cases, the second 5 mm port can be omitted. The biopsy needle is introduced into the abdomen percutaneously, and control of any biopsy site bleeding can be achieved through a single 5 mm port. 

The single-handed use of this instrument allows the surgeon to use a laparoscopic ultrasound probe at the same time. Lesions within the liver parenchyma might be biopsied in this fashion under direct ultrasound guidance. This will also allow to visualize vasculature in proximity of the lesion in order to prevent bleeding complications.

We recommend using argon coagulation to immediately control any biopsy site bleeding in these often-cirrhotic patients. The argon probe allows fast and effective control of bleeding from the biopsy site. Alternatively, a diathermy hook in spray coagulation mode (40 Watts) can be used. 

The following limitations should be taken into account before using the above-described technique. The nature of most liver lesions can be determined with the use of 4-phase multidetector CT scans or contrast-enhanced MRI. If serial or alternative modality imaging studies using the appropriate protocols fail to determine the nature of the lesion, biopsy using the described technique might be appropriate. 

## 5. Conclusion

This modification of laparoscopic liver biopsy offers a safe and efficient alternative to biopsies with a standard percutaneous liver biopsy needle. It allows access to lesions in difficult locations within the liver. Further, it frees one hand of the operating surgeon for any auxiliary manoeuvers. Minimal tissue disruption and biopsy site bleeding may contribute to decreased tumor cell seeding.
